# The anti-fibrotic effects of mesenchymal stem cells on irradiated lungs via stimulating endogenous secretion of HGF and PGE2

**DOI:** 10.1038/srep08713

**Published:** 2015-03-04

**Authors:** Li-Hua Dong, Yi-Yao Jiang, Yong-Jun Liu, Shuang Cui, Cheng-Cheng Xia, Chao Qu, Xin Jiang, Ya-Qin Qu, Peng-Yu Chang, Feng Liu

**Affiliations:** 1 Department of Radiation Oncology, The First Bethune Hospital of Jilin University, Changchun 130000, China; 2 Department of Cardiac Surgery, TEDA International Cardiovascular Hospital, Tianjin 300000, China; 3 Tianjin Allian Stem Cell Techonology CO., LTD, Tianjin 300308, China; 4 Nephrology department, China-Japan Union Hospital of Jilin University, Changchun 130000, China

## Abstract

Radiation-induced pulmonary fibrosis is a common disease and has a poor prognosis owing to the progressive breakdown of gas exchange regions in the lung. Recently, a novel strategy of administering mesenchymal stem cells for pulmonary fibrosis has achieved high therapeutic efficacy. In the present study, we attempted to use human adipose tissue-derived mesenchymal stem cells to prevent disease in Sprague-Dawley rats that received semi-thoracic irradiation (15 Gy). To investigate the specific roles of mesenchymal stem cells in ameliorating radiation-induced pulmonary fibrosis, we treated control groups of irradiated rats with human skin fibroblasts or phosphate-buffered saline. After mesenchymal stem cells were infused, host secretions of hepatocyte growth factor (HGF) and prostaglandin E2 (PGE2) were elevated compared with those of the controls. In contrast, tumour necrosis factor-alpha (TNF-α) and transforming growth factor-beta1 (TGF-β1) levels were decreased after infusion of mesenchymal stem cells. Consequently, the architecture of the irradiated lungs was preserved without marked activation of fibroblasts or collagen deposition within the injured sites. Moreover, mesenchymal stem cells were able to prevent the irradiated type II alveolar epithelial cells from undergoing epithelial-mesenchymal transition. Collectively, these data confirmed that mesenchymal stem cells have the potential to limit pulmonary fibrosis after exposure to ionising irradiation.

Radiotherapy is an essential tool for the management of thoracic malignancies. Nearly 60% of patients who suffer from non-small-cell lung cancer (NSCLC) require ionising irradiation to control the growth of their tumours^1^. However, radiotherapy-induced lung disease, including interstitial pneumonia and pulmonary fibrosis, are the main obstacles that limit the doses of radiation that can be used to control tumours^2^. Radiation-induced pulmonary fibrosis (RIPF) is a refractory disease with high morbidity and mortality rates caused by the progressive breakdown of pulmonary architecture, which ultimately results in respiratory failure^2^.

As described by Wynn^3^, the formation of an irreversible pulmonary fibrosis lesion after ionising irradiation requires four sequential processes: vascular damage-induced clotting and coagulation, infiltration by inflammatory cells, cytokine-induced activation of fibroblasts, and tissue remodelling by activated fibroblasts. The activation of fibroblasts is regarded as a key step in the pathogenesis of pulmonary fibrosis^3^. A recent study reported that transforming growth factor-beta1 (TGF-β1) plays a predominant role both in regulating the transformation of fibroblasts into myofibroblasts and in inducing type II alveolar epithelial cells (AECs) to undergo an epithelial-mesenchymal transition (EMT) into myofibroblasts by initiating the phosphorylation of Smad2/3 and reducing the expression of Dickkopf-1(Dkk-1) in a p38-dependent manner^4^,^5^,^6^. In contrast to fibroblasts, myofibroblasts, which are characterised by high expression of alpha-smooth muscle actin (α-SMA), are contractile cells that cause alveolar collapse through the production of fibres and extracellular matrix (ECM)^7^. Currently, the most useful intervention for treating RIPF is lung transplantation. However, a lack of available donated lungs and transplantation-related complications limit the wide application of this approach^2^,^3^.

Mesenchymal stem cells (MSCs), a population of multipotent cells, have performed well in the management of a variety of diseases because these cells are capable of differentiating into multiple lineages and promoting tissue regeneration. A recent study demonstrated that bone marrow-derived Flk-1^+^ MSCs had the potential to repair pulmonary tissue that had been injured by thoracic irradiation at a dose of 14 Gy; in irradiated tissue, the MSCs differentiated into functional lung cells, including endothelial and epithelial cells^8^. In other studies, gene therapy was used to treat RIPF by using bone marrow-derived MSCs as carriers of the genes for hepatocyte growth factor (HGF) or TGF-β type II receptor^9^,^10^. These genetically modified MSCs travel to injured sites and inhibit the TGF-β/Smad signalling pathway by releasing large amounts of HGF; this inhibition results in increased expression of Smad7, which interferes with the phosphorylation of Smad2/3. Similarly, TGF-β type II receptor gene-modified MSCs neutralise excessive TGF-β1 in irradiated lung tissue by expressing large quantities of TGF-β type II receptors^10^,^11^,^12^. MSCs can also be isolated from adipose tissue. Adipose-derived MSCs (Ad-MSCs) are superior to bone marrow-derived MSCs because they express high levels of Dkk-1^13^. Dkk-1 is an antagonist of the Wnt/β-catenin signalling pathway; inhibition of this pathway by Dkk-1 would reduce the pathogenesis of pulmonary fibrosis^6^. Moreover, engrafted Ad-MSCs would be able to protect themselves against Wnt/β-catenin signalling-induced differentiation into myofibroblasts through autocrine Dkk-1 protein, which would instead promote the differentiation of Ad-MSCs into functional epithelial cells^14^. Moreover, the differentiation of these cells into epithelial cells is also predicted to limit pulmonary fibrosis^15^. Thus, in the present study, we aimed to evaluate whether Ad-MSCs have anti-fibrotic potential by examining the effects of introducing Ad-MSCs from healthy human donors into a Sprague-Dawley rat model of RIPF.

## Results

### Identification of isolated Ad-MSCs *in vitro*

**Figure 1:**
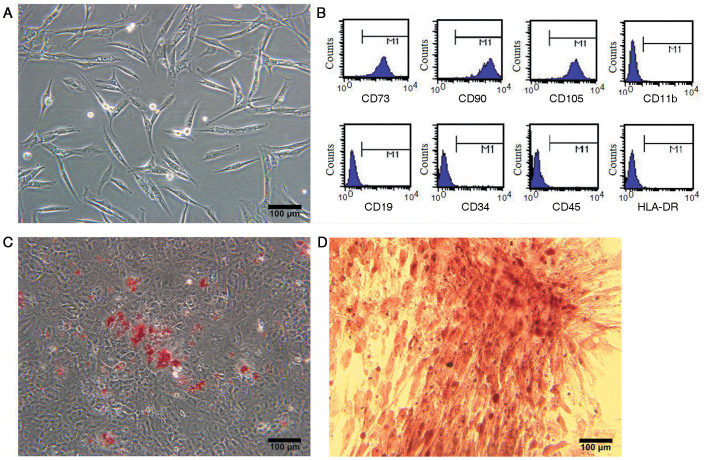
Identification of Ad-MSCs. (A)Spindle-like shape *in vitro*. (B) Phenotypic analysis by flow cytometry. (C) Adipogenic potential of Ad-MSCs: Oil Red O staining for lipid droplets in Ad-MSCs; (D): Osteogeneic potential of Ad-MSCs: Alizarin Red staining for calcium in Ad-MSCs.

*In vitro*, the Ad-MSCs were adherent and exhibited a ‘spindle-like' shape during growth (Figure 1A). In addition, fluorescence-activated cell sorting (FACS) analysis showed that the Ad-MSCs were positive for mesenchymal lineage markers, including CD73 (99.93%), CD90 (99.95%), and CD105 (99.93%), and were negative for hematopoietic lineage markers, including CD11b (0.19%), CD19 (0.14%), CD34 (0.14%), CD45 (0.38%), and HLA-DR (0.10%) (Figure 1B). Moreover, the Ad-MSCs exhibited the potential to differentiate into adipocytes and osteoblasts after 21 days in defined medium (Figure 1C and D). These results demonstrated that these cells satisfied the minimal criteria to be identified as MSCs *in vitro*^16^.

### Ionising irradiation enhances the homing efficiency of infused Ad-MSCs to injured lung tissue

**Figure 2:**
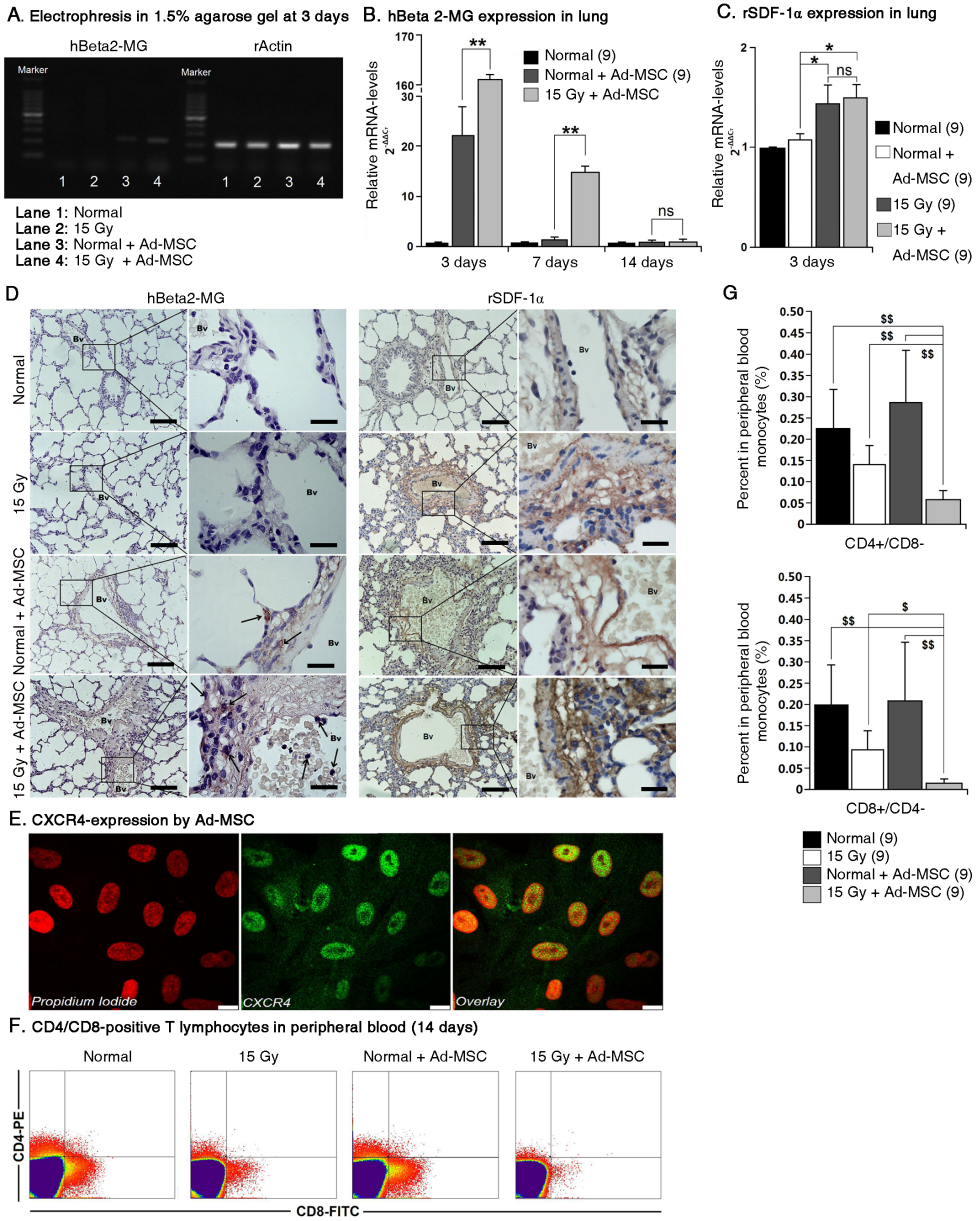
Homing of Ad-MSCs to irradiated lung. (A) Electrophesis in 1.5% agarose gel for verifying the specificity of hBeta2-MG primers to the human cells. (B) Expression of hBeta2-MG in lung tissue within 14 days post-irradiation. (C) Expression of rSDF-1α in lung tissue at 3 days post-irradiation. The expression levels of genes, including hBeta2-MG and rSDF-1α, were tested using quantitative real-time PCR. Rat Actin (rActin) was set as the internal control for determining ΔC_T_ values. Fold increases in expression were normalized to normal group by determining 2^−ΔΔCT^ values. Data shown are the mean ± S.D. of nine independent samples. This experiment was performed only once. One-way ANOVA analysis was used for comparing the differences of data among groups. *P ≤ 0.05 and **P ≤ 0.01 (significant high) *versus* the Normal + Ad-MSC group. ns: no statistical significance. (D) IHC-staining for Beta2-MG only in human cells and for SDF-1α in lung tissues at 3 days post-irradiation. Left rank: Magnification at 200×. Scale bar, 100 μm. Right rank: Magnification at 1000×. Scale bar, 20 μm. Black arrow: hBeta2-MG-positive cells. (E) IF-staining for CXCR4 in Ad-MSCs. Left: Propidium iodide for nuclear; Middle: Alexa Fluor 488 for detecting CXCR4 in Ad-MSCs; Right: Overlay. Magnification at 2000×. Scale bar, 10 μm. (F) FACS analysis for CD4^+^ or CD8^+^ T lymphocytes in peripheral blood samples at 14 days post-irradiation. (G) Percent of CD4^+^ or CD8^+^ T lymphocytes in peripheral blood monocytes (PBMCs). Upper: Percent of CD4^+^/CD8^−^ T lymphocytes; Lower: Percent of CD8^+^/CD4^−^ T lymphocytes. Data shown are the mean ± S.D. of nine independent samples. This experiment was performed only once. One-way ANOVA analysis was used for comparing the differences of data among groups. ^$^*P* ≤ 0.05 and ^$$^*P* ≤ 0.01 (significant low) *versus* the Normal group, 15 Gy group and Normal + Ad-MSC group.

It has been widely observed that MSCs are capable of homing to injured sites to facilitate tissue repair. However, Francois et al.^17^ previously reported that a low level of donor DNA from systemically infused human MSCs (accounting for ~0.06% of all MSCs) was observed in lungs from intact non obese diabetic/severe combined immunodeficient (NOD/SCID) mice, indicating that MSCs migrate to the lungs regardless of irradiation status. However, it was demonstrated that stromal cell-derived factor-1α (SDF-1α) levels were up-regulated after tissue damage, thereby forming a driving force to recruit infused MSCs to the injured sites by interacting with the C-X-C receptor 4 (CXCR4) expressed by MSCs^18^. Based on this evidence, we hypothesised that local irradiation to the right lung would enhance the homing efficiency of systemically infused Ad-MSCs to injured sites. To test this proposal, we injected 5 × 10^6^ Ad-MSCs into irradiated rats through the tail vein (15 Gy + Ad-MSC group, 27 rats). Normal (un-irradiated) rats systemically administered the same amount of Ad-MSCs were used as controls in this study (Normal + Ad-MSC group, 27 rats). In addition, to examine the products formed by primers specific for human Beta2-microglobulin (hBeta2-MG) belonging to the donor Ad-MSCs, one group of rats that did not receive Ad-MSCs and did not undergo right lung irradiation (Normal group, 27 rats) and another group of rats that did not receive Ad-MSCs but did undergo right lung irradiation (15 Gy group, 27 rats) were used to eliminate the non-specific PCR products related to hBeta2-MG amplification from rat lung mRNA. At 3, 7, and 14 days post-irradiation, 9 rats from each group were sacrificed for the following analyses. The electrophoretic analysis revealed that the primers for hBeta2-MG did not react with non-specific DNA fragments from rat lung tissue (Figure 2A). Next, using a quantitative real-time PCR method, the expression of hBeta2-MG in the injured right lungs was found to be considerably higher than that in the normal lungs at 3 days post-irradiation (a ~7.52-fold increase), indicating that the amount of Ad-MSCs in the irradiated lungs was higher than in the normal lungs (Figure 2B). To some extent, the increased number of Ad-MSCs in the irradiated lungs depended on the up-regulation of rat SDF-1α (rSDF-1α) expression in injured sites at 3 days post-irradiation and the CXCR4 expression by Ad-MSCs (Figure 2C and E). Moreover, immunohistochemical (IHC) staining for hBeta2-MG from human cells also revealed that the Ad-MSCs infiltrated towards the lung tissues after systemic infusion because the cells expressing SDF-1α were mainly located within the blood vessels (Figure 2D). However, at 7 days post-irradiation, hBeta2-MG expression in both the normal right lungs and the irradiated right lungs was decreased compared with the expression at 3 days post-irradiation. At 14 days post-irradiation, the expression of hBeta2-MG in the two groups showed no difference (Figure 2B). Moreover, the mean hBeta2-MG ΔC_T_ values (the difference between the C_T_ value for hBeta2-MG and the C_T_ value for rat Actin) in both groups were similar to those in the normal group (data not shown), indicating that the donor Ad-MSCs were probably cleared from the host lungs. Similarly, at 7 and 14 days post-irradiation, there were no differences in rSDF-1α expression between the four groups (data not shown).

Next, we aimed to determine the relationship between the clearance of Ad-MSCs and immune rejection in the host, because CD4^+^ and CD8^+^ T lymphocytes have been reported to be involved in the clearance of heterogenic MSCs^19^. From the above results, we found that the infused Ad-MSCs were cleared from irradiated lungs within 14 days. Therefore, it was essential to evaluate the variations in the number of CD4^+^ or CD8^+^ T lymphocytes after Ad-MSC infusion at this time point. Compared with the normal rats, the percentages of CD4^+^ and CD8^+^ T lymphocytes in peripheral blood samples from the infused rats with or without irradiation were decreased (Figure 2F and G). However, the percentages of CD4^+^ and CD8^+^ T lymphocytes from irradiated rats that received Ad-MSC treatment were further decreased compared with those from untreated rats (Figure 2G). These results suggest that the rapid clearance of infused Ad-MSCs from irradiated lungs is not related to the immune rejection driven by CD4^+^ and CD8^+^ T lymphocytes; this lack of immune rejection is attributed to the immune benefits of MSCs due to their low expression of MHC class I and the absence of expression of MHC class II and co-stimulatory molecules, including CD40, CD40L, CD80, and CD86^20^,^21^. In addition, the formation of immune memory might induce a major reaction by CD4^+^ or CD8^+^ T compartments after heterogenic Ad-MSC infusion^19^. Nonetheless, the explanation for Ad-MSC clearance from irradiated lungs deserves further study.

### Ad-MSC therapy protects the lungs from radiation-induced histological changes

**Figure 3:**
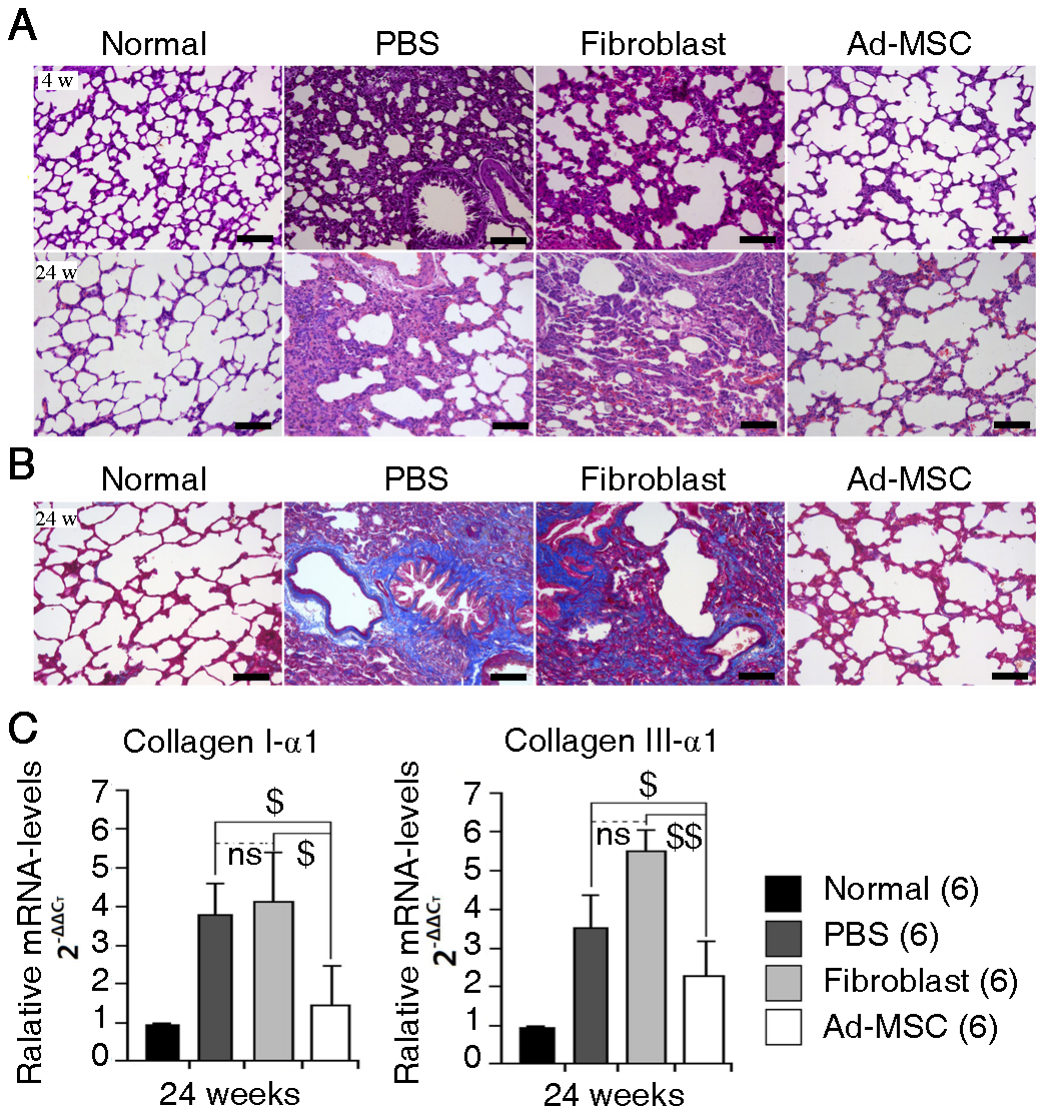
Histological changes in irradiated lungs within 24 weeks following irradiation. (A) H&E staining. Upper panel: 4 weeks post-irradiation; Lower panel: 24 weeks post-irradiation. Magnification at 100×. Scale bar, 200 μm. (B) Masson's Trichrome staining. Magnification at 200×. Scale bar, 100 μm. (C) Expressions of ECM-related genes in whole right lung at 24 weeks post-irradiation. Left: Collagen I-α1 expression; Right: Collagen III-α1 expression. The primers of Collagen I-α1 and Collagen III-α1 are designed for rat species. The expression levels of genes, including Collagen I-α1 and Collagen III-α1, were tested using quantitative real-time PCR. rActin was set as the internal control for determining ΔC_T_ values. Fold increases in expression were normalized to normal group by determining 2^−ΔΔCT^ values. The data of three samples from each group were collected in each independent experiment. The experiment was repeated for two times. For statistical analysis, the data of six samples in all two independent experiments were calculated for the value of mean ± S.D. One-way ANOVA analysis was used for comparing the differences of data among groups. ^$^*P* ≤ 0.05 and ^$$^*P* ≤ 0.01 (significant low) *versus* the PBS and Fibroblast groups. ns: no statistical significance.

This study aimed to evaluate the anti-fibrotic effects of human Ad-MSCs on irradiated lungs. First, we used 48 rats to assess the radiation-induced histological changes in lungs when treated or not treated with Ad-MSCs. A total of 36 rats received irradiation at a dose of 15 Gy to their whole right lungs. Then, 12 of the irradiated rats were infused with Ad-MSCs (Ad-MSC group). Another 12 of the irradiated rats were infused with human skin fibroblasts (Fibroblast group). A third group of 12 of the irradiated rats were infused with phosphate-buffered saline (PBS) (PBS group). Both cells and PBS were injected through the tail vein. The remaining 12 normal rats were used as controls (Normal group). At 4 and 24 weeks post-irradiation, 6 rats from each group were sacrificed. Three samples were used to evaluate histological changes, and the other 3 samples were used to extract total mRNA from the whole right lungs. When isolating the lung samples, we found that the lower lobes of the irradiated lungs were the main locations of disease (Supplementary Figure S1). In the PBS and Fibroblast groups, radiation treatment damaged lung architecture and caused lesions with massive inflammatory cell infiltration, alveolar collapse, and thickening of alveolar septa at 4 weeks and caused tissue consolidation with fewer alveoli and obvious collagen deposition at 24 weeks (Figure 3A and B). In contrast, treatment with Ad-MSCs reduced the number of infiltrating inflammatory cells within alveolar septa and attenuated collagen deposition at the irradiated site. Moreover, alveolar morphology was maintained (Figure 3A and B). To determine the degree of fibrosis in the irradiated lungs, the expression of collagen I-α1 and collagen III-α1 was used to reflect EMC formation in the irradiated lungs^22^. Compared with the normal right lungs, the irradiated lungs treated with PBS, fibroblasts or Ad-MSCs up-regulated collagen I-α1 and collagen III-α1 expression to varying degrees. However, the expression levels of these two fibrosis-related genes in the Ad-MSC group were not as high as the levels in the PBS or Fibroblast group, indicating that the development of fibrosis in the irradiated lungs was limited after infusion of Ad-MSCs (Figure 3C).

### Ad-MSC therapy reduces tissue levels of the pro-fibrotic factors TGF-β1 and TNF-α and elevates tissue levels of the anti-fibrotic factors HGF and PGE2

The above results revealed that infusion of Ad-MSCs preserves pulmonary architecture after exposure to ionising radiation at a dose of 15 Gy. Next, it was essential to determine the variations in cytokines related to the promotion and inhibition of fibrosis. To address this aim, we used 108 rats to establish a model of radiation-induced pulmonary fibrosis. The irradiated rats were divided into three groups: the PBS group, the Fibroblast group, and the Ad-MSC group. Each group contained 36 irradiated rats. Six rats from each group were sacrificed at 3 days, 1 week, 2 weeks, 4 weeks, 12 weeks, and 24 weeks post-irradiation.

**Figure 4:**
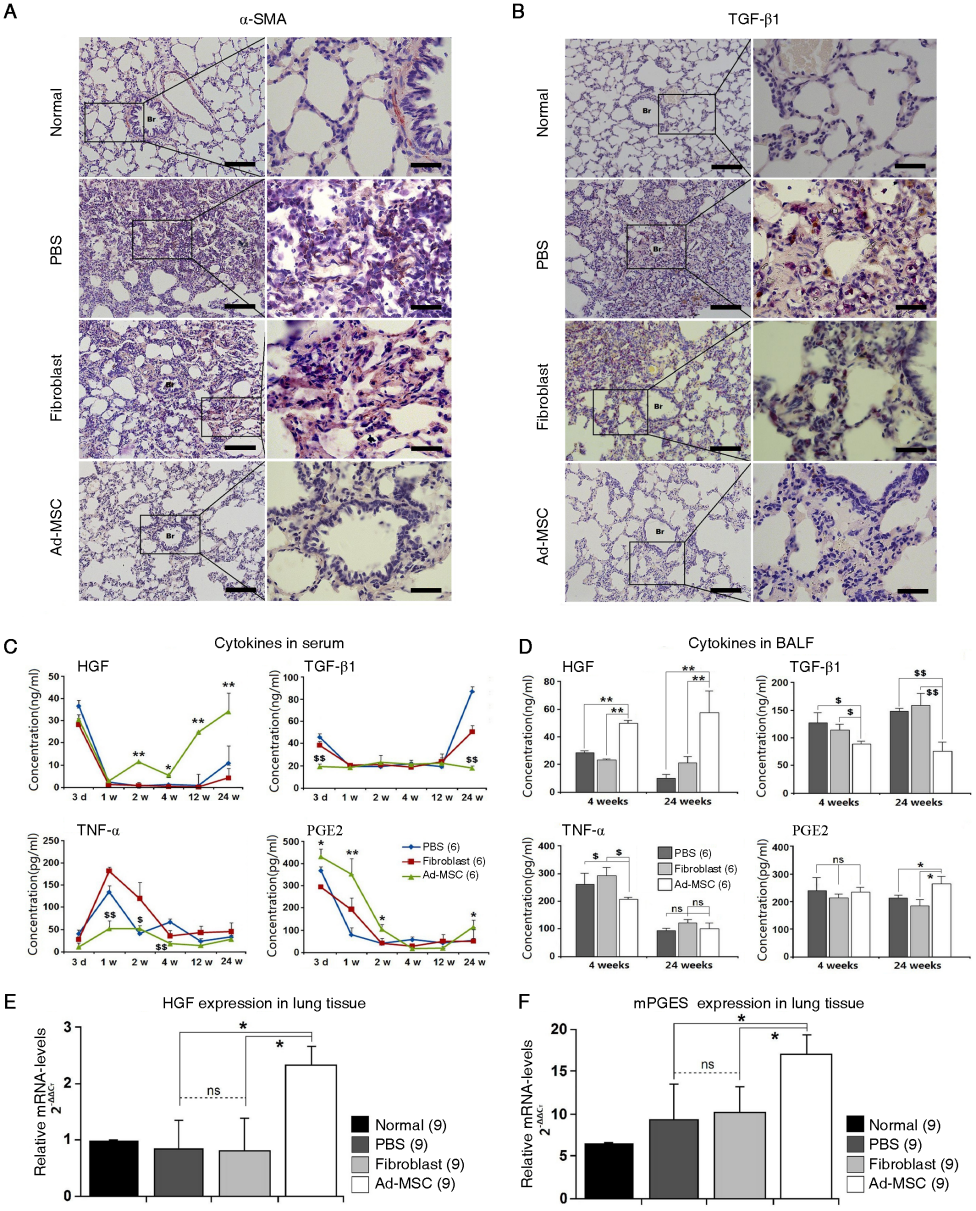
Anti-fibrotic effects of Ad-MSCs on irradiated lung. (A) IHC-staining for α-SMA. Left rank: Magnification at 200×. Scale bar, 100 μm. Right rank: Magnification at 1000×. Scale bar, 20 μm (B) IHC-staining for TGF-β1. Left rank: Magnification at 200×. Scale bar, 100 μm. Right rank: Magnification at 1000×. Scale bar, 20 μm. (C) Concentrations of cytokines in serum. Data represent the mean ± S.D. of six independent samples. This experiment was performed only once. For statistical analysis, the data of six samples were calculated for the value of mean ± S.D.One-way ANOVA analysis was used for comparing the differences of data among groups. (D) Concentrations of cytokines in bronchoalveolar lavage fluid (BALF). The data of three samples from each group were collected in each independent experiment. The experiment was repeated for twice. For statistical analysis, the data of six samples in all two independent experiments were calculated for the value of mean ± S.D. One-way ANOVA analysis was used for comparing the differences of data among groups.*P ≤ 0.05 and **P ≤ 0.01 *versus* the PBS and Fibroblast groups. ^$^*P* ≤ 0.05 and ^$$^*P* ≤ 0.01 *versus* the PBS and Fibroblast groups. It should be noted that the assays used to test for HGF, PGE2, TGF-β1, and TNF-α were for rat tissues, not human tissues.(E) Expression of HGF in irradiated lung. (F) Expression of mPGES in irradiated lung. All primers of above genes are designed for rat species. The expression levels of HGF and mPGES were tested using quantitative real-time PCR. rActin was set as the internal control for determining ΔC_T_ values. Fold increases in expression were normalized to normal group by determining 2^−ΔΔCT^values. The data of nine samples from each group were collected in only one independent experiment. For statistical analysis, the data of nine samples were calculated for the value of mean ± S.D. One-way ANOVA analysis was used for comparing the differences of data among groups. *P ≤ 0.05 and **P ≤ 0.01 (significant high) *versus* the PBS and Fibroblast groups. ^$^*P* ≤ 0.05 and ^$$^*P* ≤ 0.01 (significant low) *versus* PBS and Fibroblast groups. ns: no statistical significance.

Activated fibroblasts (termed as ‘myofibroblasts') play a major role in the development of pulmonary fibrosis by producing collagens. Thus, we first investigated whether myofibroblasts were present within injured sites through IHC-staining for α-SMA^7^. Compared with normal lung tissue, massive myofibroblasts were intermingled with alveoli in the PBS and Fibroblast groups, especially in respiratory regions. In contrast, in the Ad-MSC group, activated fibroblasts were seldom observed at 24 weeks post-irradiation (Figure 4A). Next, we investigated the expression of TGF-β1, which triggers fibroblast activation in irradiated lung tissue. As shown in Figure 4B, in contrast to results for the Ad-MSC group, 24 weeks after treatment with PBS or fibroblasts, TGF-β1 was primarily expressed within thickened alveolar septa. The secretion of TGF-β1 in serum exhibited two peaks, with the highest concentrations reaching 87.6 ± 3.7 ng/ml in the PBS group and 53.1 ± 4.1 ng/ml in the Fibroblast group at 24 weeks post-irradiation (Figure 4C). However, the levels of TGF-β1 in the Ad-MSC group were not markedly altered by ionising irradiation; the levels of TGF-β1 at 3 days and 24 weeks post-irradiation were significantly lower in this group compared with the PBS and Fibroblast groups (*P* ≤ 0.01). Similarly, the levels of tumour necrosis factor-alpha (TNF-α) in serum were substantially lower after treatment with Ad-MSCs during the first 4 weeks post-irradiation (*P* ≤ 0.05). In contrast to the variability in TGF-β1 and TNF-α levels in serum, the serum levels of HGF in the Ad-MSC group were constantly elevated throughout the period from 1 to 24 weeks post-irradiation (Figure 4C). Moreover, compared with the PBS and Fibroblast groups, the levels of PGE2 in serum were considerably increased in the Ad-MSC group at 2 and 24 weeks post-irradiation (*P* ≤ 0.05). In addition, the levels of these factors in bronchoalveolar lavage fluid (BALF) were tested at 4 and 24 weeks post-irradiation; these time points were chosen to reflect the variability in the levels of these factors within injured tissues (Figure 4D). Compared with the PBS and Fibroblast groups, infusion of Ad-MSCs considerably increased the levels of HGF in BALF (*P* ≤ 0.01) but reduced the levels of TGF-β1 and TNF-α (*P* ≤ 0.05). Infusion of Ad-MSCs also markedly increased the levels of PGE2 in serum and BALF during the 24-week post-irradiation period compared with the levels in the other two groups (*P* ≤ 0.05). Levels of the anti-inflammatory factor interleukin-10 (IL-10) were measured in both serum and BALF but were not significantly different among the groups (data not shown). The above results indicate that treatment with Ad-MSCs may limit the activation of fibroblasts by elevating the release of HGF and PGE2, while host production of TGF-β1 and TNF-α is simultaneously reduced.

Next, we aimed to determine the origins of anti-fibrotic effectors, including HGF and PGE2, in irradiated lung tissues. First, the expression levels of genes, including HGF, cyclooxygenase-1 (COX-1), cyclooxygenase-2 (COX-2), membrane-associated prostaglandin E synthase (mPGES), and cytosolic prostaglandin E synthase (cPGES), were evaluated using real-time PCR assays. At 3 days post-irradiation, the HGF and mPGES genes showed up-regulated expression in the Ad-MSC group (Figure 4E and F), whereas the infusion of Ad-MSCs did not alter the expression of the other three genes in this group compared with the other groups. Similarly, the expression of the five genes listed above did not show any differences among the groups, indicating that AECs (type I and type II AECs) were not the cellular origins of HGF and PGE2 following Ad-MSC treatment (data not shown). Based on the immunogold-silver staining technique, previous results also indicated that AECs were not positive for COX-1 or COX-2^23^. IHC-staining for HGF and PGES in lung sections revealed that the main cellular locations of PGES and HGF were the bronchial epithelial cells and the stromal cells surrounding the blood vessels and the bronchial epithelial cells, respectively (Supplementary Figure S2), as demonstrated previously^23^,^24^. The increased level of HGF in Ad-MSC-treated lungs probably leads to further elevation of PGE2 synthesis in bronchial epithelial cells^24^.

### Ad-MSC therapy protects irradiated type II AECs from undergoing EMT

**Figure 5:**
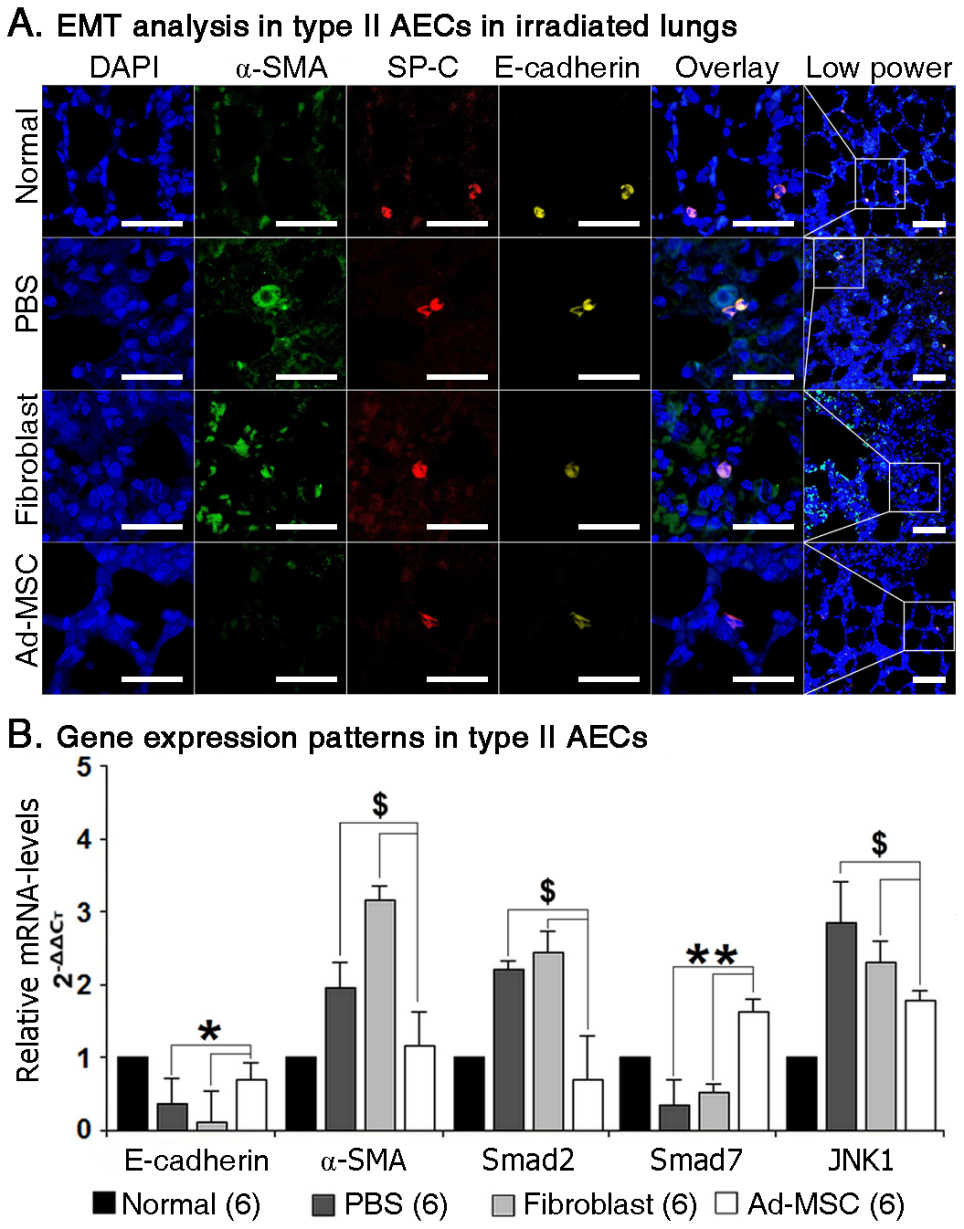
EMT in type II AECs. (A) Confocal imaging using Z-stacks technology at 24 weeks post-irradiation. The thickness of the section was set as 15 μm. The thickness between frames was set as 0.25 μm. The SP-C marker were used for identifying type II AECs, co-staining with α-SMA and E-cadherin in type II AECs. Magnification at 1500× in the first five ranks. Scale bar, 200 μm. Magnification at 630× in the last one rank. Scale bar, 200 μm. (B) Gene expression patterns in type II AECs. Type II AECs were isolated at 24 weeks post-irradiation using microbeads. The expression levels of genes, including E-cadherin, α-SMA, Smad2, Smad3, Smad7, JNK1 and JNK2, were tested using quantitative real-time PCR. All primers of these genes are designed for rat species. rActin was set as the internal control for determining ΔC_T_ values. Fold increases in expression were normalized to normal type II AECs by determining 2^−ΔΔCT^ values. The data of three samples from each group were collected in each independent experiment. The experiment was repeated for two times. For statistical analysis, the data of six samples in all two independent experiments were calculated for the value of mean ± S.D. One-way ANOVA analysis was used for comparing the differences of data among groups. *P ≤ 0.05 and **P ≤ 0.01(significant high) *versus* PBS and Fibroblast groups. ^$^*P* ≤ 0.05 (significant low) *versus* PBS and Fibroblast groups.

The mechanisms by which ionising irradiation itself, together with the subsequent release of TGF-β1, triggers EMT in type II AECs have recently been elucidated^4^,^25^,^26^. Type II AECs undergoing EMT lose their polarity and become detached with up-regulated α-SMA expression and down-regulated E-cadherin expression^22^. In the present study, we observed that α-SMA-positive myofibroblasts were predominantly located on alveolar luminal surfaces (Figure 4A). Based on this finding, EMT in type II AECs was analysed at 24 weeks post-irradiation by performing immunofluorescence (IF) staining for SP-C, a marker for type II AECs, as well as α-SMA and E-cadherin. Unlike normal type II AECs, type II AECs from the PBS and fibroblast groups expressed α-SMA, suggesting that EMT occurred in these cells (Figure 5A). In contrast, the lack of α-SMA expression also revealed that EMT in type II AECs was commonly inhibited by the delivery of Ad-MSCs (Figure 5A). However, the number of type II AECs was not markedly increased by Ad-MSC infusion (Figure 5A). Next, type II AECs were isolated from irradiated lungs using immunomagnetic beads, and the expression of EMT-related genes in isolated cells was compared among the groups. As shown in Figure 5B, after Ad-MSC infusion, the expression of E-cadherin and Smad7 was considerably up-regulated (P ≤ 0.05), whereas that of Smad2 and JNK1 was considerably down-regulated (P ≤ 0.05). There were no significant differences in the expression of Smad3 and JNK2 among the groups. These results indicate that Ad-MSCs have the potential to protect type II AECs from radiation-induced EMT.

## Discussion

In this study, our results revealed that systemic infusion of Ad-MSCs had anti-fibrotic effects on irradiated lungs. After Ad-MSC infusion, the architecture of the irradiated lung was preserved, as represented by a lack of transformation of fibroblasts into myofibroblasts and reduced ECM formation within injured sites. It is essential to first explain the pathogenesis of RIPF before analysing the exact roles played by Ad-MSCs in protecting lungs against radiation-induced fibrosis.

The process of fibrotic lesion formation in irradiated lungs can generally be classified into three stages, namely, a latent phase (≤1 week post-irradiation), a pneumonitic phase (2 ~ 16 weeks post-irradiation), and a fibrotic phase (≥24 weeks post-irradiation)^26^. However, the detailed biochemical reactions involved in the pathogenesis of RIPF are complicated. Apart from the ionising irradiation contributing to the development of pulmonary fibrosis by directly activating EMT in type II AECs, fibrotic formation is largely driven by the abnormal release of fibrosis facilitators, such as TGF-β, CTGF, TNF-α, IFN-γ, IL-1β, and IL-6, after exposure to high doses of ionising radiation^3^,^25^,^27^. Previous data confirmed that whole-chest irradiation with a dose of 12 Gy induced high expression of TGF-β in the lungs of C57BL/6 mice^26^. Recently, the mechanisms by which TGF-β1, a member of the TGF-β superfamily, promotes fibrosis have been well studied using the rodent model of bleomycin-induced idiopathic pulmonary fibrosis (IPF); these mechanisms include the differentiation of fibroblasts into myofibroblasts and induction of type II AECs to undergo EMT to form myofibroblasts^5^,^19^. Moreover, the EMT-inducing effect of TGF-β1 on type II AECs can be strengthened by TNF-α^4^. In this study, after treatment with PBS or fibroblasts, the levels of TGF-β1 in serum exhibited peaks in the latent phase (3 days post-irradiation) and the fibrotic phase (24 weeks post-irradiation). In addition, the levels of TNF-α in the serum and BALF from these two groups increased to varying degrees over a period of 24 weeks post-irradiation. In the milieu of high concentrations of TGF-β1 and TNF-α, resident fibroblasts were activated, and EMT commonly occurred in type II AECs, along with up-regulated α-SMA, Smad2, and JNK1 expression. Following these changes, activated fibroblasts would acquire proliferative potential and secrete excessive ECM into interstitial tissue, which ultimately results in tissue remodelling^28^. In contrast, the levels of TGF-β1 and TNF-α in both serum and BALF were not markedly altered over 24 weeks after Ad-MSC infusion following ionising radiation, and the levels of these two cytokines were low compared with the levels in the PBS and fibroblast groups. Consequently, the architecture of the irradiated lungs in the Ad-MSC group was well preserved and did not exhibit the massive activation of fibroblasts and marked ECM formation observed in the other two groups.

Although the therapeutic effects of human Ad-MSCs on RIPF were demonstrated, the exact roles played by Ad-MSCs deserve further discussion. In the present study, we found that the Ad-MSCs homed to injured lungs within 3 days post-infusion. It is reasonable to speculate that Ad-MSCs facilitate tissue repair by secreting multiple cytokines and differentiating into tissue-specific cells, including functional lung cells. However, in our opinion, some cytokines derived from human MSCs are not capable of providing rat cells with the essential signals necessary to initiate their proliferative and/or anti-apoptotic responses, which can be attributed to species variation. Moreover, previous results have indicated that systemically delivered MSCs are rapidly cleared within 4 days post-transplantation in the irradiated host^9^. In this study, we observed that the Ad-MSCs disappeared from the irradiated lungs within 14 days post-infusion; however, there is a lack of evidence to demonstrate that the clearance of the Ad-MSCs was driven by host CD4^+^ or CD8^+^ T cells. Despite the apparent clearance of the cells, the long-lasting anti-fibrotic effects persisted for a period of 24 weeks post-irradiation, suggesting that the indirect roles played by infused Ad-MSCs may help the host to increase its response to foreign stimuli. Recently, cumulative evidence has suggested that heterogenic transplantation using human MSCs triggers the intrinsic repair process in a variety of diseases, such as stroke, Parkinson's disease, inflammatory bowel disease, and radiation-induced injuries, by increasing the levels of endogenous growth factors and/or anti-inflammatory effectors^29^,^30^,^31^,^32^. In this study, the anti-fibrotic benefits of Ad-MSC infusion may be attributable, at least in part, to the stimulation of endogenous secretion of anti-fibrotic factors, including HGF and PGE2, with the concomitant stabilisation of TGF-β1 and TNF-α expression at low levels. This action is also referred to as the ‘paracrine effect' of MSCs on tissue repair^33^.

HGF and PGE2 are common cytokines involved in tissue repair. In addition to facilitating angiogenesis, HGF is regarded as an anti-fibrotic facilitator, because it attenuates bleomycin-induced EMT in type II AECs by increasing intracellular levels of Smad7 upon binding to c-Met and up-regulates the expressions of matrix metalloproteinases-1, -3, and -9 in injured sites in a PI3K/Akt/p70-dependent manner to promote apoptosis of myofibroblasts^11^,^34^,^35^. With respect to the anti-fibrotic effects of PGE2, accumulating evidence has suggested that treatment with PGE2 inhibits TGF-β1-induced activation and fibroblast proliferation, thereby reducing the production of α-SMA and collagens by elevating intracellular cAMP levels; there is also evidence that treatment with PGE2 induces apoptosis in myofibroblasts by increasing the activity of the PTEN protein, which blocks the PI3K/Akt signalling pathway^7^,^36^,^37^,^38^,^39^,^40^. Moreover, Bauman et al.^41^ observed that PGE2 derived from IMR-90 cells, a human embryonic lung fibroblast cell line, exhibits a peak in secretion when cultures of these cells are supplemented with HGF at a concentration of 6 ng/ml. In the present study, we found that the mean HGF serum level at 24 weeks after Ad-MSC infusion was 33.8 ng/ml. With such a high level of HGF induced by Ad-MSC infusion, the level of PGE2 in BALF was thus markedly increased at 24 weeks post-irradiation.

As described above, interstitial pneumonia lesions precede fibrosis in irradiated lungs^26^. Moreover, it is well known that MSCs attenuate inflammatory responses by increasing the number of regulatory T cells and/or enhancing the levels of anti-inflammatory cytokines, such as IL-10^31^. Previous work demonstrated that bone marrow-derived MSCs can attenuate the inflammatory response in irradiated lungs by upregulating IL-10 expression in the latent phase and downregulating TNF-α, IFN-γ, IL-1β, and IL-6 expression^9^,^10^. However, the present data only confirmed that Ad-MSC infusion can mitigate inflammation in the host by decreasing the TNF-α level without causing any obvious alteration in IL-10 levels. To some extent, this finding can be attributed to the fact that the number of CD4^+^/CD25^+^/Foxp3^+^ regulatory T cells, which secrete IL-10^41^, was not increased in the irradiated lungs within the first 2 weeks after Ad-MSC infusion (Supplementary Figure S3).

In this study, we did not observe an increased number of type II AECs at 24 weeks post-irradiation. However, a previous study demonstrated that massive amounts of proliferative type II AECs, double-positive for SP-C and Ki-67, were found in injured lungs at 6 months post-irradiation. Moreover, the action of EMT in type II AECs is involved in the development of pulmonary fibrosis induced by irradiation^22^. However, in another study, it was demonstrated that the action of EMT in type II AECs did not contribute to pulmonary fibrosis induced by bleomycin^42^.The ionising irradiation approach was used to establish the pulmonary fibrosis model for our analysis. Although the type II AECs did not significantly increase in number at 24 weeks post-irradiation regardless of whether they received PBS, fibroblast, or Ad-MSC treatment, it was still observed that Ad-MSCs were capable of protecting irradiated type II AECs from undergoing EMT. Ionising irradiation of the lung and bleomycin delivery appeared to have different impacts on pulmonary fibrosis, especially on whether EMT of type II AECs is involved in fibrotic formation.

In terms of EMT, a previous study reported that the transcription factors, such as Smad2 and JNK1, would upregulate their expressions in human A549 cells, when treating with TGF-β^22^. Moreover, persist expressions of these two factors were also engaged in promoting the EMT process in A549 cells^22^. The present results showed that, after receiving Ad-MSC treatment, the irradiated type II AECs expressed high levels of Smad7 and low levels of Smad2 and JNK1 comparing to PBS or Fibroblast group, and maintained their expression-patterns of E-cadherin and α-SMA as in normal type II AECs. In spite of this, we still lacked evidences for explaining the mechanisms by which Ad-MSC treatment maintained the epithelial phenotypes of irradiated type II AECs, because changes in expressions of above transcription factor-downstream genes, which engaged in post-transcriptional processing of E-cadherin/α-SMA genes and in post-translational modification of E-cadherin/α-SMA proteins, were not evaluated. This is a limitation in the present study.

In the murine model of bleomycin-induced pulmonary fibrosis, bone marrow-derived ‘fibrocytes', which are positive for CXCR4, were reported to participate in the formation of fibrotic lesions by differentiating into myofibroblasts and producing type I collagen^43^. Previous data suggest that SDF-1 expression is increased at injury sites, and this response could be further strengthened after MSC infusion^18^. Based on this evidence, the Ad-MSCs were predicted to promote pulmonary fibrosis after irradiation because the interaction between SDF-1 and CXCR4 was hypothesised to initiate the migration of ‘fibrocytes' from bone marrow to injured sites. However, in the present study, we found that the SDF-1α gene expression in irradiated lungs was increased only at 3 days post-irradiation. Thereafter, the expression of SDF-1α quickly decreased to normal levels at 7 days post-irradiation. Whether ‘fibrocytes' participating in fibrotic formation in irradiated lungs warrants further investigation. Nevertheless, the benefits of Ad-MSC infusion in RIPF were demonstrated in the present study.

In summary, our results revealed that the infusion of human Ad-MSCs was beneficial in protecting lung tissue from radiation-induced fibrosis, and that this protection was largely the result of increased levels of endogenous HGF and PGE2 after Ad-MSC infusion. Although the Ad-MSCs were cleared from the injured lung shortly after infusion, the long-lasting therapeutic effects persisted. In conclusion, human Ad-MSCs constitute a novel mechanism by which to trigger intrinsic repair actions by the host, especially in heterogenic transplantation.

## Methods

### Animals

Male Sprague-Dawley rats, weighting 150 ~ 180 g, were provided by the Laboratory Animal Center of the Academy of Military Medical Sciences (Beijing, China). All animals were used in accordance with animal care and use guidelines, and all animal experiments were approved by our institution's animal care and use committee.

### Model of RIPF

Rats were anaesthetized with an intraperitoneal injection of 10% chloral hydrate (0.6 ml/100 g) and were placed on a platform to receive a single fraction dose of 15 Gy. The irradiated field covered the whole right chest from the collarbone to the arc of the rib (semi-thoracic irradiation). X-rays were produced using an RS-2000 Pro Biological Irradiator (Rad-Source, Suwanee, GA, USA) with a real-time dose rate of 1.5 Gy/minute.

### Cell preparation

Adipose tissue was donated by a healthy, female human. Before MSCs were isolated, the donor was informed of the procedures, and her approval was obtained. MSCs were isolated following the procedure described in our previous study^44^. In brief, fragments of adipose tissue were digested using 0.2% Type IV collagenase (Gibco, Grand Island, NY, USA) at 37°C for 40 minutes. The resulting cell suspension was passed though a 70-μmstrainer (BD Bioscience, Franklin Lakes, NJ, USA) to remove any undigested mass. The filtered suspension was centrifuged at a force of 233 × *g* for 5 minutes, and the supernatant was removed. Then, cells were resuspended with complete medium containing 10% FBS (Gibco) and 90% DMEM-LG/F12 (Gibco). The cells were added to flasks and cultured at 37°C in a humidified atmosphere containing 5% CO_2_. Passage 3 Ad-MSCs were used in this study. Adult human skin-derived fibroblasts (ATCC number: PCS-201-012^TM^, MD, USA) and PBS were used in control experiments. Within 2 hours after ionizing radiation, 5 × 10^6^ Ad-MSCs in 1.5 ml of PBS, 5 × 10^6^ fibroblastsin 1.5 ml of PBS and 1.5 ml of PBS alone were infused through the tail vein. Rats that were treated with PBS were enrolled in the PBS group, and rats that were treated with fibroblasts were enrolled in the Fibroblast group. Finally, rats that were treated with Ad-MSCs were enrolled in the Ad-MSC group.

### Identifying phenotypes of Ad-MSCs

Mouse anti-human CD11b-PE, CD19-FITC, CD34-FITC, CD45-PE, CD73-PE, CD90-PE, CD105-PE and HLA-DR-PE were used to analyze the phenotypes of Ad-MSCs. Mouse IgG1-fluorescein isothiocyanate (FITC) and IgG1-phycoerythrin (PE) were set as isotype controls. All antibodies were purchased from BD Bioscience, and the data were analyzed using FACSEALIBUR equipment (BD Bioscience).

### Differentiation assay

The procedures for inducing Ad-MSCs into adipocytes and osteocytes in vitro were in accordance with that reported previously^45^. Briefly, Ad-MSCs were added to a 6-well plate and allowed to grow overnight. When Ad-MSCs were adherent, the complete medium in 4 wells was replaced with defined media: adipogenesis differentiation medium in 2 wells and osteogenesis differentiation medium in 2 wells. The Ad-MSCs were induced in these defined media for 15 ~ 21 days at 37°C in a humidified atmosphere containing 5% CO_2_, following the instructions provided by the manufacturer (Invitrogen Inc., Carlsbad, CA, USA). Fat droplets and calcium were detected using Oil Red O and Alizarin Red staining, respectively.

### ELISA

After a rat was anesthetized, 1 ml of peripheral blood was extracted from the heart (Supplementary Video S1). A serum sample was harvested by centrifugation at a force of 12,000 × *g* at 4°C. To prepare for BALF sample collection, after shutting the left bronchus, the right lung was washed using 5 ml of pre-warmed 37°C PBS; samples were collected from 3 aspirations. Thereafter, the fluid was centrifuged at a speed of 12,000 × *g* at 4°C to remove the cell fragments. All samples were preserved at −80°C before use. To test cytokine levels, a Rat/Mouse HGF enzyme-linked immunosorbent assay (ELISA) Kit (R&D Systems, Inc., Minneapolis, MN, USA), a Prostaglandin E2 Parameter Assay Kit (R&D Systems), a Rat TGF-β1 ELISA Kit (eBioscience, San Diego, CA, USA), a Rat IL-10 ELISA Kit (eBioscience) and a Rat TNF-α ELISA Kit (eBioscience) were used in this study. All experimental procedures were in accordance with the instructions provided by manufacturers. The cytokine-levels in serum from each six samples were tested only once in one independent experiment. The data were calculated for the value of mean ± S.D. And the cytokines-levels in BALF from each three samples were tested twice in two independent experiments. The data including total six samples were calculated for the value of mean ± S. D.

### Histological analysis

Lung samples were harvested at 4 weeks and 24 weeks post-irradiation, and paraffin sections were made. Sections were used for hematoxylin and eosin (H&E), Masson's Trichrome (MT), IHC and IF staining. Hematoxylin, eosin, iron-hematoxylin, acid fuchsin, phosphomolybdotungstic acid and aniline blue were purchased from Sigma-Aldrich Corporation (St. Louis, MO, USA). The primary antibodies for IHC and IF were α-SMA (Abcam, Cambridge, MA, USA), TGF-β1 (Abcam), Human Beta2 Microglobulin (Abcam), CXCR4 (Abcam), HGF (Abcam), PGES (Abcam), E-cadherin (Santa Cruz Biotechnology, Santa Cruz, CA, USA), SDF-1α (Santa Cruz Biotechnology), AQP5 (Santa Cruz Biotechnology) and SP-C (Santa Cruz Biotechnology). The secondary antibodies Alexa Fluor 488 (Invitrogen), Alexa Fluor 594 (Invitrogen) and Alexa Fluor 633 (Invitrogen) were used for IF-staining. 4',6-diamidino-2-phenylindole DAPI (Invitrogen) and propidium iodide (Invitrogen) was used for counter staining to detect nuclei. Horseradish peroxidase (HRP)-conjugated goat anti-rabbit/mouse IgG (H + L) secondary antibodies (Abcam) and AEC single solution (Invitrogen) were used in IHC-staining. Besides, Rabbit IgG (Abcam), Mouse IgG2α (Abcam) and Mouse IgG1 (Abcam) were used as isotype controls (Supplementary Figure S4). A TCS SP5-II microscope (Leica, Germany) was used for confocal imaging. ADM 2500 microscope (Leica, Germany) was used for bright field imaging.

### CD4^+^ or CD8^+^ T lymphocytes

The PBMCs were isolated using the method reported previously^46^. Briefly, 5 ml of peripheral blood sample was harvested using heart puncture (Supplementary Video S1). Then, the blood sample was mixed with PBS at a ratio of 1:1. After that, the mixture was added into the centrifuge tube of 15 ml Ficoll-Paque Plus (GE Healthcare, USA), and centrifugated at a speed of 2000 rpm without acceleration for 20 min. The cells in buffy coat were collected into a new tube. Wash the cells using PBS for 2–3 times and add Mouse anti-Rat CD4-PE antibody and Mouse anti-Rat CD8-FITC antibody. Mouse IgG1κ-FITC antibody and Mouse IgG2α-PE antibody were set as isotype controls. All antibodies were purchased from eBioscience company. The amounts of antibodies were determined by total cells in tubes. The staining procedure followed the instructions provided by manufacture. The cells were analyzed using Beckman MoFlo XDP equipment (Beckman Coulter, Inc. Brea, CA, USA). The data of nine samples in each group, indicating the expressions of beta2-MG and SDF-1α, were calculated for the mean ± S.D. This experiment was performed only once.

### CD4^+^/CD25^+^/Foxp3^+^ regulatory T lymphocytes

Cells were harvested from the irradiated right lung using mechanical digestion and 0.25% Trypsin/EDTA (Invitrogen) for 5 minutes at 37°C on a shaker. Single-cell suspensions were prepared by sequential filtering with a 70-μm strainer (BD Bioscience), a 40-μm strainer (BD Bioscience) and an 800mesh strainer. The single-cell suspension was then centrifuged at a force of 200 × *g* for 5 minutes, and the supernatant was discarded. After washing the cells with PBS 2–3 times, the CD4(+)CD25(+)Foxp3(+) regulatory T lymphocytes were stained using a Rat Regulatory T Cell 3-Color Flow Kit (R&D Systems) according to the manufacturer's protocol. Thereafter, the cells were analyzed using FACSEALIBUR equipment (BD Bioscience). This experiment was performed only once.

### Real-time PCR

The procedure for isolating a single cell from the irradiated lung was as described above. After preparation of a single-cell suspension using DMEM/F12 (Gibco), the SP-C antibody (Santa Cruz Biotechnology) and AQP5 antibody (Santa Cruz Biotechnology) was respectively added to the sample at a concentration of 1:50 (w/v), and samples were incubated for 30 minutes on ice to mark the type II AECs and type I AECs, seperately. Next, any unconjugated antibody remaining was removed by washing the sample twice with PBS and resuspending with DMEM/F12 (Gibco). Then, goat anti-Rabbit IgG Microbeads (MiltenyiBiotec, Teterow, Cologne, Germany) and goat anti-mouse IgG Microbeads (MiltenyiBiotec) were added to the sample at a ratio of 1:4 (v/v),and samples were incubated for 20 minutes on ice. The cells were sorted using a magnetic column (MiltenyiBiotec) and were then collected and counted. A total of 1 × 10^6^ sorted cells were immersed in 1 ml of TRIzol reagent (Invitrogen) to isolate total mRNAs from type I AECs and type II AECs. Besides, 100 mg of freshly isolated lung tissue was immersed in 1 ml of TRIzol reagent (Invitrogen) to extract total mRNAs from irradiated lung. One microgram of total RNA was used for first-strand cDNA synthesis using an RT-PCR Kit (Takara-bio Inc., Shiga, Japan). Then, total cDNA was amplified over 40 cycles in a system containing SYBR Green I TaqMan probes (Roche, Basel, Switzerland). The primers used in this study are listed in Supplementary Table S1. All primers were synthesized by Invitrogen, Inc. The data of nine samples in each group, indicating the expressions of beta2-MG, SDF-1α, HGF, COX-1, COX-2, mPGES and cPGES, were calculated for the mean ± S.D. This experiment was performed only once. For testing the expressions of Collagen I-α1, Collagen III-α1, E-cadherin, α-SMA, Smad2, Smad3, Smad7, JNK1 and JNK2, data of total six samples from each group in two separate experiments were used for calculating the value of mean ± S.D.

### Statistical analysis

The data were analyzed using SPSS (Statistical Package for the Social Sciences) 17.0 software (SPSS Inc., Chicago, IL, USA) and are shown as the mean ± S.D. Data among groups were compared by One-way ANOVA test. Statistical significance was defined as *P* ≤ 0.05.

## Author Contributions

L.H.D. and P.Y.C. wrote the main manuscript text. P.Y.C. and F.L. conceived and designed the experiments. Y.Y.J. prepared figures 4 ~ 5 and Supplementary Figure S3. C.C.X. prepared figures 1 and 3 and Supplementary Figure S1. Y.J.L. and S.C. prepared figures 2 ~ 5 and Supplementary Figure S2 and S4. C.Q. and X.J. analyzed all the data. Y.Q.Q. provided reagents and materials. All authors reviewed the manuscript.

## Supplementary Material

Supplementary Information Heart puncture

Supplementary Information Supplementary information
