# Supplementary material for: The anti-fibrotic effects of mesenchymal stem cells on irradiated lungs via stimulating endogenous secretion of HGF and PGE2
Source: Sci Rep. 2015 Mar 4;5:8713. doi: 10.1038/srep08713 (PMC4348621; doi:10.1038/srep08713)
Supplement: Supplementary Information [file srep08713-s2.pdf]

Title : The anti-fibrotic effects of mesenchymal stem cells on irradiated lungs via stimulating endogenous secretions of HGF and PGE2

Author list: Li-Hua Dong, Yi-Yao Jiang, Yong-Jun Liu, Shuang Cui, Cheng-Cheng Xia, Chao Qu, Xin Jiang, Ya-Qin Qu, Peng-Yu Chang* and Feng Liu

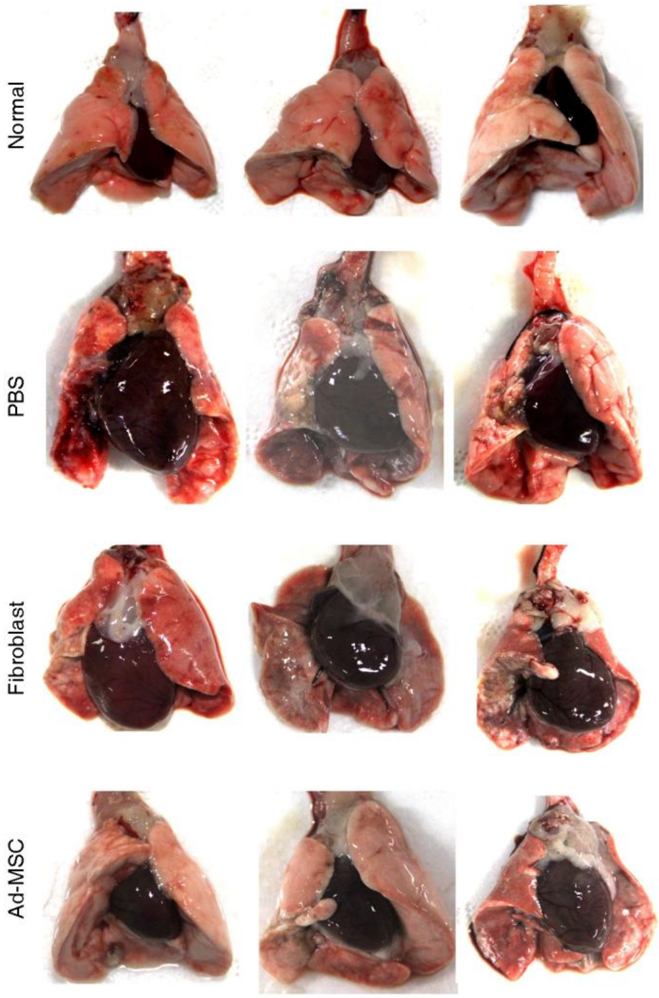

Supplementary Figure S1

Lung samples isolated at 24 weeks post-irradiation.

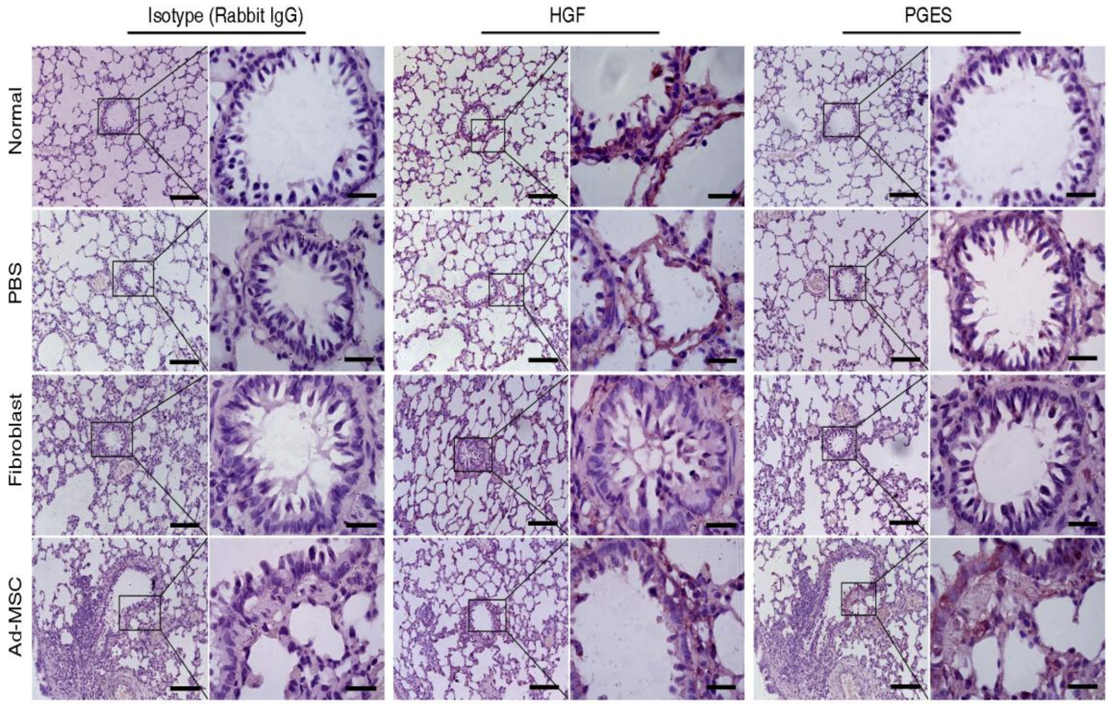

Supplementary Figure S2

Identifying the origins of HGF and PGE2 in lung tissue at 3 days post-irradiation. (Left) IHC-staining for isotype control of HGF and PGES(Rabbit IgG), Left rank: Magnification at 200 ×; Scale bar, 100 µm. Right rank: Magnification at 1000 ×; Scale bar, 20 µm. (Middle) IHC-staining for HGF, Left rank: Magnification at 200 ×; Scale bar, 100 µm. Right rank: Magnification at 1000 ×; Scale bar, 20 µm.(Right) IHC-staining for PGES, Left rank: Magnification at 200 ×; Scale bar, 100 µm. Right rank: Magnification at 1000 ×; Scale bar, 20 µm.

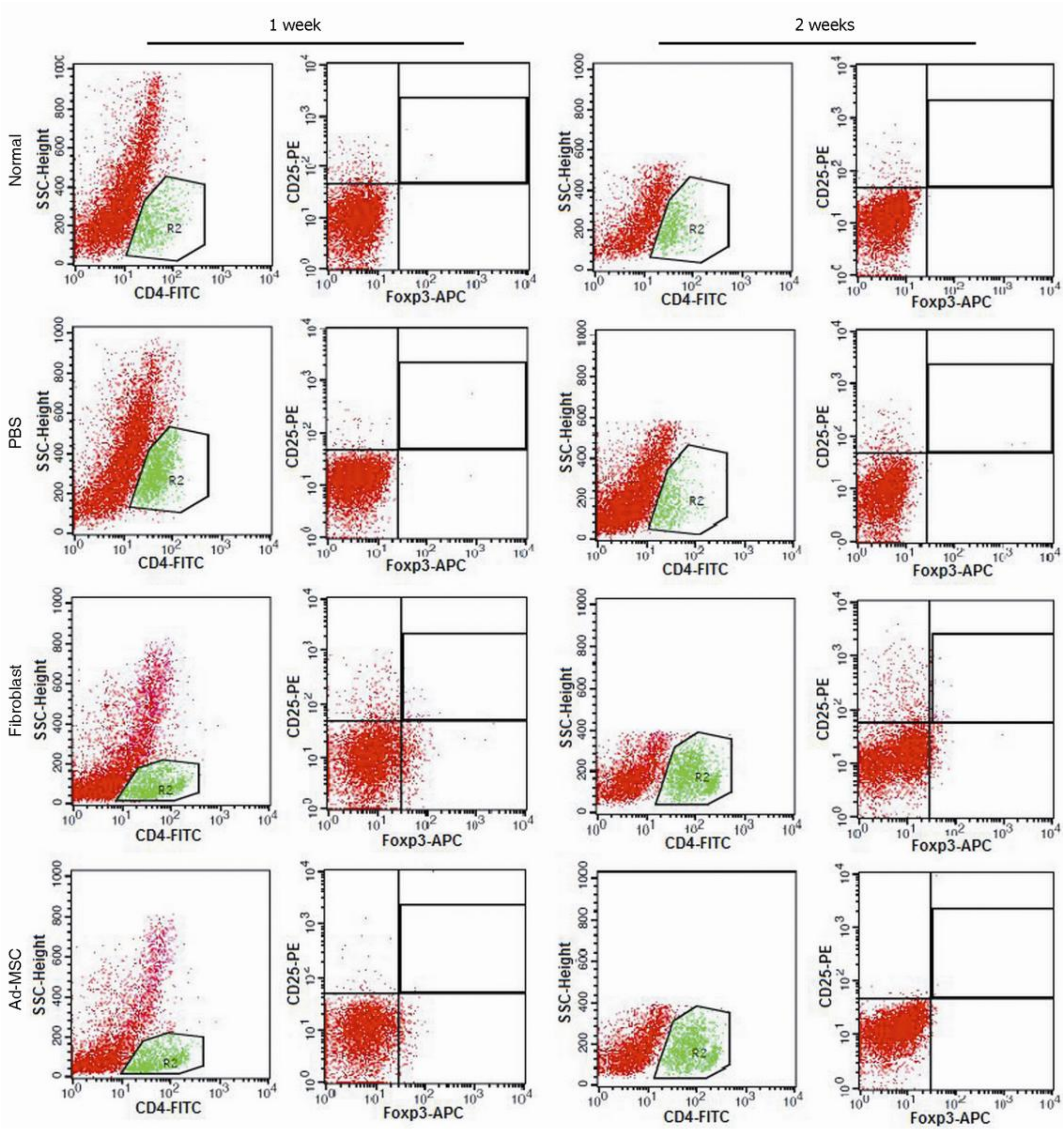

Supplementary Figure S3

FACS analysis for $CD4^{+}/CD25^{+}/Foxp3^{+}$ regulatory T lymphocytes in irradiated lung. Left two panels: 1 week post-irradiation. Right two panels: 2 weeks post-irradiation. Goat IgG-PE and Goat IgG-APC were used as isotype controls.

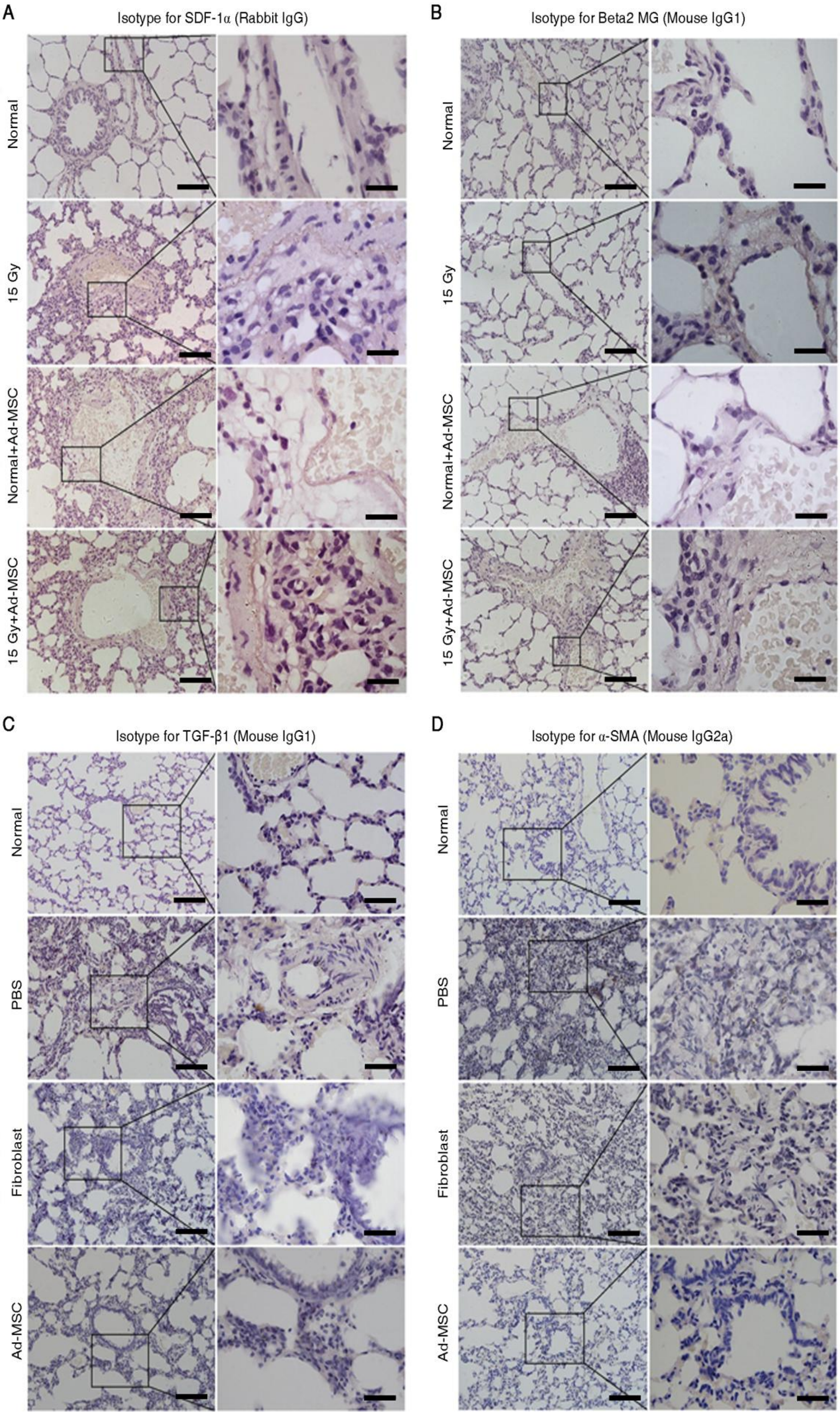

A
Isotype for SDF-1α (Rabbit IgG)
Normal
15 Gy
Normal+Ad-MSC
15 Gy+Ad-MSC
B
Isotype for Beta2 MG (Mouse IgG1)
Normal
15 Gy
Normal+Ad-MSC
15 Gy+Ad-MSC
C
Isotype for TGF-β1 (Mouse IgG1)
Normal
PBS
Fibroblast
Ad-MSC
D
Isotype for α-SMA (Mouse IgG2a)
Normal
PBS
Fibroblast
Ad-MSC

Supplementary Figure S4

IHC-staining for Mouse IgG1, Mouse IgG2a and Rabbit IgG. (Upper -Left) IHC-staining for isotype control of SDF-1α (Rabbit IgG) . Left rank: Magnification at 200 ×; Scale bar, 100 μm. Right rank: Magnification at 1000 ×; Scale bar, 20 μm. (Upper-Right) IHC-staining for isotype control of human Beta2-MG (Mouse IgG1). Left rank: Magnification at 200 ×; Scale bar, 100 μm. Right rank: Magnification at 1000 ×; Scale bar, 20 μm. (Lower-Left) IHC-staining for isotype control of TGF-β1(Mouse IgG1), Left rank: Magnification at 200 ×; Scale bar, 100 μm. Right rank: Magnification at 1000 ×; Scale bar, 20 μm. (Lower-Right) IHC-staining for isotype control of α-SMA (Mouse IgG2α), Left rank: Magnification at 200 ×; Scale bar, 100 μm. Right rank: Magnification at 1000 ×; Scale bar, 20 μm.

Supplementary Table S1. Primer sequences and product sizes.

Primer	Sequence (5’ → 3’)	Product size (bp)
E-cadherin	Forward TTGGCACACTGATGGTGAGG<br>Reverse GATTGGGCAACTCAGGTCCA	206
α-SMA	Forward TGCTGACAGGATGCAGAAGG<br>Reverse AGAACTGAAGGCGCTGATCC	303
Smad2	Forward ATGAGCTTCGTGAAGGGGTG<br>Reverse CTCCACTGCTGACGGACTTT	177
Smad3	Forward CTGCAGTGCCGCTATCCTG<br>Reverse TTCACGTTCTGCGTGGTGAT	339
Smad7	Forward AGTCTCGGAGGAAGAGGCTG<br>Reverse GGGAAGCAGGGACAGAAGAT	268
JNK1	Forward TGACGCCTTACGTGGTAACTC<br>Reverse AAAGCACATCGGGGAACAGT	294
JNK2	Forward TGAAAGACCAGCCTTCAGCAC<br>Reverse ACAGACAAGTGCGCCATCTG	193
SDF-1α	Forward ACGGTCTTGAACTACTGGCG<br>Reverse GGAGGCTTACAGCACGAAAC	183
Beta2-MG	Forward AGATGCCGCATTTGGATTGG<br>Reverse CCTCTAAGTTGCCAGCCCTC	221
HGF	Forward GTGCATCAGAAACAAGGGCT<br>Reverse TGGCACTTGATGCCACTCTT	231
COX-1	Forward CCCAATGTCCAGCAAGGGTT<br>Reverse ATAGGAATGTGGCGGCTCCC	159
COX-2	Forward TTTCAATGTGCAAGACCCGC<br>Reverse CAACACAGGAATCTTCACAAATGG	269
mPGES	Forward GCCCTACAGATCCTCTGGGA<br>Reverse GGCTCAGAGATCCACACACC	181
cPGES	Forward GCGCCCCTTTTCCTACACTT<br>Reverse TCGGTCGTACCACTTTGCAG	268
Collagen I-α1	Forward GCCTCTGCAACAAATCCCCA<br>Reverse CATGTGTGGCCGATGTTTCC	125
Collagen III-α1	Forward AATGGAGCAAAGGGAGAGCC<br>Reverse ACCCCTTTCTCCTGGGTTTC	150
β-actin	Forward CACCCGCGAGTACAACCTTC<br>Reverse CCATACCCACCATCACACCC	206

Supplementary Video S1

Isolating the blood sample using heart puncture method
